# A Practical Approach for Ischemic Preconditioning Intervention in Sports: A Pilot Study for Cuff Thigh Occlusion Pressure Estimation Based on Systolic Blood Pressure

**DOI:** 10.5114/jhk/186064

**Published:** 2024-04-15

**Authors:** Géssyca T. de Oliveira, Hiago L. R. Souza, Eduardo O. Prazeres, Bernardo P. Bernardes, Stephen D. Patterson, Rhaí André Arriel, Gustavo Bittencourt Camilo, Rodrigo Hohl, Anderson Meireles, Moacir Marocolo

**Affiliations:** 1 Department of Biophysics and Physiology, Exercise Physiology Performance—EXPPER, Federal University of Juiz de Fora, Juiz de Fora, Brazil.; 2 Centre for Applied Performance Science, St. Mary’s University, London, United Kingdom.; 3 Department of Anatomy, Federal University of Juiz de Fora, Juiz de Fora, Brazil.

**Keywords:** ischemia, reperfusion, arterial pressure, tourniquet

## Abstract

For the ischemic preconditioning (IPC) intervention, the accuracy of the protocol is paramount for mediating its possible ergogenic effects. However, the lack of standardization and widespread use of arbitrary cuff pressures (ranging from 130 to >300 mmHg) have been predominantly observed, potentially affecting the results and compromising the reproducibility of findings. Thus, the purpose of this study was to determine an appropriate cuff pressure during IPC. Seventeen healthy male participants were enrolled in the study. Anthropometric measurements were initially conducted, followed by systolic and diastolic blood pressure measurements. Subsequently, we determined the individual thigh occlusion pressure (TOP) for the right leg using a hand-held Doppler device. Based on these findings, we developed an estimation equation for TOP, considering the current brachial systolic blood pressure (SBP) values. We then conducted a retrospective analysis of its capacity to mediate occlusion. We observed the ability to estimate TOP using the equation (p = 0.01; ES: 0.86), presenting ~6% superiority in absolute values for occlusion compared to direct measurement (TOP equation: 169.9 ± 9.1; TOP direct measured: 161.2 ± 11.1). However, TOP estimation was insufficient to produce complete occlusion in two out of 17 subjects (11.8%). In conclusion, the estimation of TOP incorporating SBP values may offer a valid and practical means for cuff administration during IPC protocols with potential to minimize adverse effects and maximize its positive effects.

## Introduction

Ischemic preconditioning (IPC) consists of brief periods of blood flow occlusion followed by reperfusion (Sharma et al., 2015). Its effects are largely described in the literature, showing reductions in tissue damage during long periods of ischemia (Stokfisz et al., 2017) or improvements in exercise, sports performance and recovery (Marocolo et al., 2023). For IPC intervention, a pneumatic cuff is usually administrated in the proximal region of arms or thighs and the applied cuff pressure alternates between high (during occlusion phases) and low or none (during reperfusion cycles) (Cocking et al., 2018). Pneumatic cuff administration is also used in clinical settings for preventing bleed, for example during orthopaedic surgeries and in these cases, a sustained high cuff pressure is applied during long times, without any release (Addison et al., 2003).

Considering the necessity of occluding the blood flow, for short or long periods, a cuff pressure above systolic blood pressure should be applied to ensure complete arterial occlusion (Committee, 2007; Sharma and Salhotra, 2012). In this context, there is a lack of standardization and safety guidelines about using pneumatic cuffs and it is common to find a large range of cuff applied pressures, from studies using IPC acutely with 130 mmHg cuff pressure (O'Brien and Jacobs, 2021) and orthopedic surgeries applying more than 350 mmHg cuff pressure for more than 60 min, continuously (Zhang et al., 2017).

Excessive cuff pressure administration may lead to tissue damage, with the extent of damage directly related to cuff pressure (Olivecrona et al., 2013). This risk remains significant even during short periods of use. Given that cuff administration is integral to various clinical and sports practices, including blood flow restriction training (Jarosz et al., 2023), IPC (Marocolo et al., 2019), surgeries (Zhang et al., 2017), among others, employing minimal effective pressure becomes crucial to mitigate injuries and facilitate cuff intervention (Committee, 2007; Sharma and Salhotra, 2012). Also, a huge discomfort is reported by subjects when an exacerbated cuff pressure is applied on the upper or lower limbs during IPC intervention (Sharma et al., 2014).

However, the accessibility of tools for arterial occlusion pressure measurements could be an obstacle to the effective and secure implementation of these methodologies. While the utilization of a Medical Ultrasound Doppler as a gold standard is far from being a viable practice, portable Dopplers or devices measuring systolic blood pressure are very easy to use, and constitute applicable and inexpensive measurements. Furthermore, portable Dopplers have a high reliability and validity in precisely detecting blood flow occlusion (Vehrs et al., 2023, 2024), which could facilitate a practical and easy use of this type of equipment.

Thus, the aims of this study were 1) to determine the individual thigh arterial blood flow occlusion pressure (TOP) using a hand-held Doppler device, and 2) to propose an equation that would incorporate systolic blood pressure for accurate estimations of TOP.

## Methods

### Participants

Seventeen healthy men participated in this study (Table 1), with no history of cardiovascular, metabolic or pathological conditions and not taking any medications. All volunteers were instructed to abstain from consuming coffee, tea, and alcohol for 24 h and to avoid vigorous exercise for 48 h preceding each intervention. The experimental procedure received approval from the Federal University of Juiz de Fora (protocol code 4.120.625, date of approval: 29 June 2020) and adhered to the principles outlined in the Declaration of Helsinki. Moreover, prior to data collection, all volunteers signed an informed consent form.

**Table 1:** Demographic, anthropometric, and hemodynamic characteristics of participants (n = 17).

Variables	Mean ± SD	Minimum	Maximum
Age (years)	27.2 ± 7.4	21.0	44.0
Weight (kg)	80.2 ± 8.9	63.0	98.0
Height (cm)	176.2 ± 6.9	162.0	186.0
BMI (kg•m^−2^)	25.8 ± 2.6	20.8	33.9
Thigh circumference (cm)	57.4 ± 5.4	48.0	66.0
SBP (mmHg)	125.6 ± 9.1	111.0	140.0
DBP (mmHg)	72.6 ± 7.6	53.0	82.0
MAP(mmHg)	90.3 ± 6.9	72.7	98.0
TOP(mmHg)	161.2 ± 11.1	140.0	180

Data are expressed as mean ± standard deviation. BMI, body mass index; SBP, systolic blood pressure; DBP, diastolic blood pressure; MAP, mean arterial pressure; TOP, thigh arterial occlusion pressure

### Measures

Brachial systolic (SBP) and diastolic (DBP) blood pressure were measured using an automatic cuff (Contec™, model ABPM50; 19.6 x 68 cm). TOP was determined using a pneumatic cuff (Riester®, Germany, 96 length x 13 cm width). Sound signs of pulse presence were assessed with a hand-held bidirectional vascular Doppler (Medpeg^2^ model OV-200l, Ribeirão Preto, Brazil) placed on the anterior tibial artery.

### Design and Procedures

Upon arrival at the laboratory, anthropometric measurements were conducted encompassing body height, body mass, skinfold thickness, and thigh circumferences. Following a 5-min rest interval in a supine position, SBP and DBP were measured. Finally, TOP was determined in the lower body.

### Brachial Systolic and Diastolic Blood Pressure

After a 5-min supine rest position, the cuff was positioned at the axillary midline portion on the brachial artery, with the lower edge of the cuff positioned 2–3 cm above the antecubital fossa for the arm. Blood pressure was measured in duplicate and values were averaged before analysis.

### Thigh Arterial Occlusion Pressure Determination (TOP)

After 5-min supine rest, a pneumatic cuff was positioned at the proximal region of the right thigh. The occlusion protocol involved incremental cuff inflation, starting at 0 mmHg, and increasing by 20 mmHg up to 100 mmHg followed by 10 mmHg increments until blood flow was no longer detected. Each pressure range was held for 30 s to stabilize the flow. The pulse at the limb was detected using a hand-held bidirectional vascular Doppler device positioned at the anterior tibial artery. Audible signals from the Doppler probe indicated if the pulse was present. TOP was recorded where no audible signal was detected. Previous evidence demonstrated high reliability of hand-held Doppler devices in measuring TOP (Vehrs et al., 2023, 2024). Additionally, in a prior laboratory analysis, we found TOP measurements of 156.3 ± 9.2 and 153.8 ± 7.4 mmHg for a hand-held and a medical ultrasound doppler, respectively, with a non-significant mean difference between them of 2.5 ± 7.1 mmHg (95%CI = −8.4 to 3.4). The analyses showed that the mean difference between these devices was significantly similar to zero (*p* = 0.351), without proportion bias (*p* = 0.522), that is, the values were evenly distributed above and below the mean difference line.

### Equation for TOP Estimative

After calculating the difference between the measured TOP and SBP of volunteers as well as their respective confidence intervals (CIs), the upper limit of 99% of the CI was used to formulate an equation to estimate the occlusion pressure, by adding the value of SBP to the upper limit value. The analysis aimed to assess the feasibility of using this equation as a reliable indicator for estimating occlusion pressure, with the 99% CI serving as a criterion to gauge the precision of this estimate. The occlusion pressure equation was then retrospectively checked to determine whether this estimation was comparable to the TOP observed by the hand-held Doppler device.

### Statistical Analysis

Statistical analyses were executed using GraphPad Prism Software (version 8. San Diego. CA. USA). Descriptive statistics were conducted to provide a comprehensive overview of the data. To ensure the validity of the findings, an assessment of the normality of the data related to demographic anthropometric and hemodynamic variables and the blood flow was carried out using the Shapiro-Wilk test. A paired *t*-test was conducted to analyze differences between the average TOP estimated by the equation and the average TOP observed by the hand-held Doppler device utilized to cease the blood flow. Effect sizes (ES) for the paired *t*-test were determined with Cohen’s *d*. The magnitude of ES was arbitrated as small (0.2), medium (0.5) or large (0.8) (Cohen, 1992). The level of significance was set at *p* < 0.05.

## Results

Individual values for all measured variables are presented in Table 2. Mean SBP and TOP directly measured were 125.6 and 161.2 mmHg, respectively. None of the participants were hypertensive or presented any discrepancies in hemodynamic variables. Calculated mean difference between TOP and SBP was 36.6 ± 12.6 mmHg with 99%CI of 26.9 to 44.3.

**Table 2:** Individual values for systolic blood pressure, arterial occlusion pressure and thigh arterial blood flow occlusion pressure.

Participants	SBP (mmHg)	TOP (mmHg)	Difference TOP-SBP	Estimated TOP (by equation)
1	120	140	20	164.
2	125	150	25	169.3
3	120	160	40	164.3
4	130	160	30	174.3
5	132	160	28	176.3
6	130	170	40	174.3
7	120	150	30	164.3
8	120	160	40	164.3
9	133	170	37	177.3
10	111	180	69	155.3*
11	125	150	25	169.3
12	137	170	33	181.3
13	140	180	40	184.3
14	125	160	35	169.3
15	115	170	55	159.3*
16	140	160	20	184.3
17	112	150	38	156.3
Mean ± SD	125.6 ± 9.1	161.2 ± 11.1	35.6 ± 12.3	169.9 ± 9.1
CV (%)	7.22%	6.89%	34.53%	5.33%

SBP: systolic blood pressure; TOP: thigh occlusion pressure; CV: Coefficient of variation; * denotes TOP estimation was insufficient to produce arterial occlusion

Considering TOP and SBP difference with the 99%CI upper limit, we elaborated an equation to estimate TOP. The equation for TOP estimation for the lower body is as follows:

*TOP_estimation_= SBP*+44.3

where: TOP_estimation_ = estimation of thigh arterial occlusion pressure; SBP = systolic arterial pressure.

In a retrospective analysis, statistical significance was observed (*p* = 0.01; ES: 0.86) between TOP estimative by equation (169.9 ± 9.1; range: 155.3 to 184.3) and TOP measured directly by the hand-held Doppler device (161.2 ± 11.1; range: 140.0 to 180.0), with TOP estimative presenting ~6% of superiority in absolute values for occlusion. The utilization of the TOP estimative equation showed 88.2% of efficacy, being capable of occluding the blood flow in 15 out of the 17 evaluated participants (Table 2).

## Discussion

Our main aim was to determine TOP by incorporating SBP into an equation to estimate the minimal required pressure applied to the cuff. We observed a nearly 90% success rate with this approach for TOP determination, which may enhance the use of occlusion pressure determination.

Different methods can be used to measure TOP, such as medical ultrasound or pulse oximetry. However, instruments of high costs, complexity, and training requirements (e.g., medical ultrasound) may render extrapolation outside controlled laboratory environments unfeasible (Vehrs et al., 2024). Additionally, methods such as pulse oximetry, while presenting as valid and reliable devices for measuring TOP (Brekke et al., 2020; Zeng et al., 2019), may vary in accuracy depending on factors such as the rate of absorption of the light-emitting electrodes of the equipment. These factors, in turn, are influenced by several variables including probe positioning, cold temperature, skin pigmentation, excessive movement, poor perfusion, or fingernail polish (Zeng et al., 2019). Therefore, incorporating an equation to estimate TOP would allow for a more practical and accessible tool to achieve minimal occlusion pressure and enhance practice.

The pressure applied in occlusion protocols for both upper and lower limbs in IPC or clinical protocols is typically set arbitrarily for all individuals (Hughes et al., 2017; Marocolo et al., 2019; Xiaolin et al., 2023; Zhang et al., 2017), without considering inter-subject variability. One of the criticisms of the IPC methodology has been the lack of standardization in cuff pressure (O'Brien and Jacobs, 2021; Salvador et al., 2016). This non-standardization raises concerns about the efficacy of the experimental protocol in achieving a complete interruption of the blood flow, irrespective of whether IPC demonstrates effects on performance. Otherwise, the use of too much pressure could lead to considerable discomfort, injuries and withdrawal from volunteers, mainly in the sports context when the subject is not under anesthesia.

It could be highlighted that cuff width directly influences the minimal required pressure for blood flow occlusion, demonstrating an inversely proportional relationship between the two (Loenneke et al., 2012). We used a suitable wide cuff (13 cm) for the experimental protocol, achieving an average value of 161.2 mmHg for TOP determination. The estimation of blood flow occlusion pressure was also described in the literature (Loenneke et al., 2015), based on a proposal with a great number of variables (i.e., limb circumference, SBP and DBP). However, those authors used a narrow cuff of 5-cm wide, commonly applied for blood flow restriction training protocols, due to the necessity of joint movements. In this context, it is important to note that a wider cuff requires much less pressure to promote occlusion than a narrower one (Loenneke et al., 2012).

Although narrow cuffs are used in a different context, when the subject needs to move joints, in the IPC scenario, there is no need to use a narrow cuff, as greater mobility is unnecessary since the occlusion occurs prior to the commencement of the exercise. Since we used a 13-cm wide cuff, we were able to achieve a total occlusion with less than 200 mmHg, while the other experiment required more than 300 mmHg of cuff pressure (Loenneke et al., 2015). This marks a considerable difference of approximately 125 mmHg compared to the cuff used in the present study.

Another consideration is the potential discomfort that may arise from applying excessively high pressure to the subject's limb. After achieving occlusion, there is no requirement to further increase pressure. Moreover, the substantial discomfort associated with elevated pressure is reflected in high scores on the subjective pain scale (Sharma et al., 2014). Such high scores may be interpreted negatively by volunteers, potentially triggering a nocebo effect. Hence, the possibility of utilizing an equation to estimate TOP further enhances the application of IPC as an ergogenic aid. Considering our findings, the proposed equation presents itself as a more practical and accessible approach for assessing TOP in IPC protocols. The simplicity and ease of using this estimation method make it an interesting choice for practical applications.

Finally, the measurements of TOP were taken in the supine position, in line with most IPC studies (Arriel et al., 2019; Crisafulli et al., 2011; de Souza et al., 2021). Consequently, these results may not directly be applied to a seated or a standing rest position, considering potential orthostatic changes in arterial pressure. Additionally, our study included only one visit to the laboratory to implement the cuff inflation protocol and measure TOP. Given this, researchers should consider the day-to-day variability in TOP measurements when designing longitudinal protocols. Lastly but not least, the present work is a pilot study where we examined solely one cuff width on the lower limb only without considering limb anatomy (circumference, fat, and muscle tissue). Hence, extrapolation for other cuff widths or upper limbs needs precautions.

## Conclusions

Our findings indicate that the estimation of TOP incorporating SBP values offers a starting point for more practical means for cuff administration during ischemic preconditioning protocols without cuff pressure exacerbation. Researchers are advised to consider employing this approach for new studies using compression maneuvers with pneumatic cuffs.
